# Reducing Stock-Outs of Life Saving Malaria Commodities Using Mobile Phone Text-Messaging: *SMS for Life* Study in Kenya

**DOI:** 10.1371/journal.pone.0054066

**Published:** 2013-01-17

**Authors:** Sophie Githinji, Samwel Kigen, Dorothy Memusi, Andrew Nyandigisi, Agneta M. Mbithi, Andrew Wamari, Alex N. Muturi, George Jagoe, Jim Barrington, Robert W. Snow, Dejan Zurovac

**Affiliations:** 1 Malaria Public Health Cluster, KEMRI-Wellcome Trust–University of Oxford Collaborative Programme, Nairobi, Kenya; 2 Division of Malaria Control, Ministry of Public Health & Sanitation, Nairobi, Kenya; 3 Management for Sciences of Health, Nairobi, Kenya; 4 Medicines for Malaria Venture, Geneva, Switzerland; 5 SMS for Life, Novartis AG, Basel, Switzerland; 6 Centre for Tropical Medicine, Nuffield Department of Clinical Medicine, University of Oxford, Oxford, United Kingdom; 7 Center for Global Health and Development, Boston University School of Public Health, Boston, Massachusetts, United States of America; Tulane University School of Public Health and Tropical Medicine, United States of America

## Abstract

**Background:**

Health facility stock-outs of life saving malaria medicines are common across Africa. Innovative ways of addressing this problem are urgently required. We evaluated whether SMS based reporting of stocks of artemether-lumefantrine (AL) and rapid diagnostic tests (RDT) can result in reduction of stock-outs at peripheral facilities in Kenya.

**Methods/Findings:**

All 87 public health facilities in five Kenyan districts were included in a 26 week project. Weekly facility stock counts of four AL packs and RDTs were sent via structured incentivized SMS communication process from health workers’ personal mobile phones to a web-based system accessed by district managers. The mean health facility response rate was 97% with a mean formatting error rate of 3%. Accuracy of stock count reports was 79% while accuracy of stock-out reports was 93%. District managers accessed the system 1,037 times at an average of eight times per week. The system was accessed in 82% of the study weeks. Comparing weeks 1 and 26, stock-out of one or more AL packs declined by 38 percentage-points. Total AL stock-out declined by 5 percentage-points and was eliminated by the end of the project. Stock-out declines of individual AL packs ranged from 14 to 32 percentage-points while decline in RDT stock-outs was 24 percentage-points. District managers responded to 44% of AL and 73% of RDT stock-out signals by redistributing commodities between facilities. In comparison with national trends, stock-out declines in study areas were greater, sharper and more sustained.

**Conclusions:**

Use of simple SMS technology ensured high reporting rates of reasonably accurate, real-time facility stock data that were used by district managers to undertake corrective actions to reduce stock-outs. Future work on stock monitoring via SMS should focus on assessing response rates without use of incentives and demonstrating effectiveness of such interventions on a larger scale.

## Introduction

Universal and continuous availability of recommended artemisinin-based combination therapy (ACT) is a critical prerequisite for the effective management of clinical malaria [1]. However, across Africa stock‐outs of ACTs are frequently reported resulting in compromised access to effective treatment, suboptimal case‐management practices and increased childhood mortality [2], [3], [4]. As traditional interventions strengthening supply chain for anti-malarial drugs and improvements of the routine logistic information systems continue to be applied [5] there is need for innovative ways of addressing public health emergencies such as stock-outs of life saving therapies. With the rapid growth and adoption of mobile phone technology in Africa, new approaches involving use of text messages are increasingly recognized as an innovative way of transmitting data from the periphery of the health system to control managers at the district and central level [6], [7]. Such reporting systems should however demonstrate high reporting rates of accurate data able to initiate responses to data signals which result in improved delivery of health services [8].

In Kenya, artemether-lumefantrine (AL) was introduced to health facilities as the recommended first-line treatment for uncomplicated malaria in 2006 [9]. The current Kenyan National Malaria Strategy [10] and Monitoring and Evaluation Plan [11] specify that by 2013, all health facilities should have AL continuously in stock. Furthermore, all facilities should stock malaria rapid diagnostic tests (RDT) reflecting a recent change of diagnostic policy towards universal parasitological testing [12]. However, recent surveys across the country report high levels of AL stock-outs, and malaria diagnostics in the limited areas where universal parasitological diagnosis and RDTs have been implemented [13], [14].

In this paper we report on the adaptation of an *SMS for Life* system, first developed and piloted in Tanzania [15], to test whether visibility of stock data transmitted by SMS could result in reduction of AL and RDT stock-outs in Kenya.

## Methods

### Description of Study Sites

The study was undertaken at all 87 public health facilities in five rural Kenyan districts (Machakos, Msambweni, Ijara, Manga and Vihiga) from August 2011 to February 2012. The districts were purposively chosen by the Division of Malarial Control (DOMC) to include areas of different malaria endemicities; supply chain mechanisms; and hard to reach areas with potentially lower mobile network coverage. The Kenya Medical Supply Agency (KEMSA) is the major supplier of AL in all five districts and distributes commodities from the central stores directly to the health facilities. The frequency of distribution is quarterly to peripheral facilities and bimonthly to hospitals. Depending on the facility level and its administrative location, facilities receive AL either based on quantities ordered (pull system) or predetermined quantities (push system). With respect to RDTs, all districts apart from Ijara had received RDTs prior to the beginning of the project.

### Description of *SMS for Life* System

**Figure 1:**
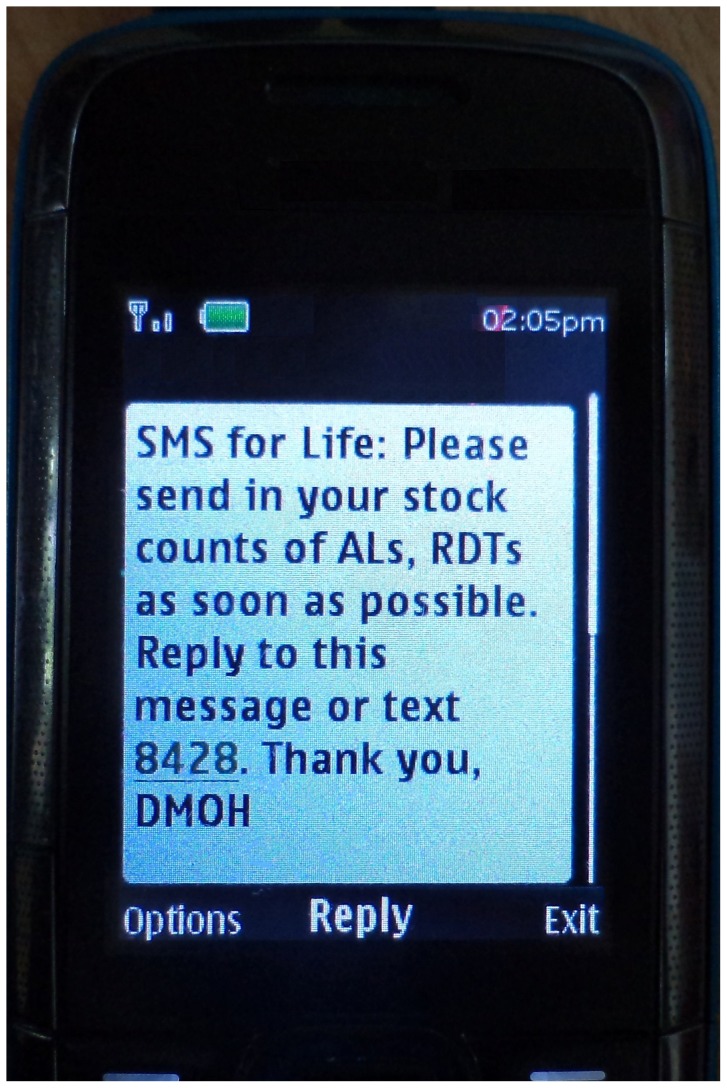
A mobile phone displaying the stock request message sent to registered health workers.

*SMS for Life* system is a web-based real-time data reporting application accessed through the internet and secured via a username and password. The system used *Mango* platform developed by *Greenmash* company (http://www.greenmash.com/). The system consists of two-components; an SMS management tool and a web-based reporting tool. The first component, SMS management tool, stores the mobile phone number of one health worker from each registered health facility. The system uses a free short code number which enables registered health workers to receive a weekly data request text message and send a reply of stocks available at their health facility at no cost. The stock data request message was sent to all registered health workers every Thursday at 2 pm (Figure 1).

Registered health workers counted each of the four weight-specific AL packs and individual RDT tests and composed the stock reply message using system specific letter codes (A = AL 6, B = AL 12, C = AL 18, D = AL 24 and T = RDT) followed by the respective number of packs or tests available at the facility. The health workers sent the reply message to the free short code number and got a confirmatory text message indicating that data had been received. In case of a formatting mistake, the system sent an error message asking the health worker to check and resend the message. On Friday at 2 pm, the SMS system sent an automatic reminder message to all registered health workers who had not responded to the data request message sent the previous day. The SMS system credited airtime value of 50 Kenyan shillings (0.6USD) to health worker’s mobile phone if the SMS data were sent before 5 pm on Friday. Messages received after 5 pm on Friday were considered late and no credit was awarded. However, late messages were accepted until 1 pm on Thursday of the following week. Beside the stock component, the same communication process was followed for an additional surveillance text message reported weekly on Mondays. Results of the surveillance component will be presented separately.

**Figure 2:**
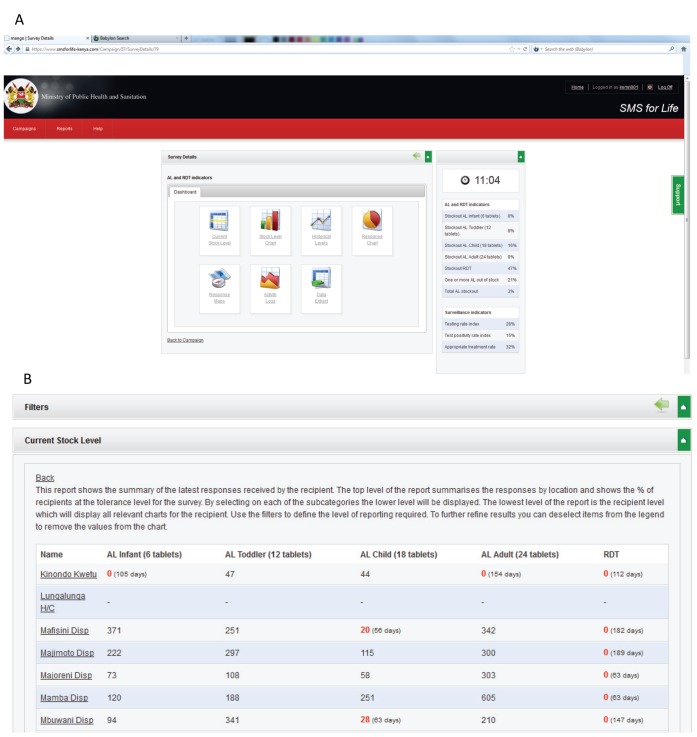
Screenshots of *SMS for Life* web-based reporting tool. (A) *SMS for Life* System dashboard. (B) Display of health facilities showing stock levels and stock-out alerts.

The second component, web-based reporting tool, captured the data sent via SMS by the registered health workers and made it available in real time through the internet to designated members of the District Health Management Team (DHMT) and to DOMC officers at the national level. The website provided current and historical data on AL and RDT stock levels from each health facility and aggregated at the district level; Google mapping of district health facilities with stock levels overlays and stock-out alerts; SMS statistics (received messages, error messages and their timing); system use statistics and a data extraction tool (Figure 2A). Using a username and password, DHMT members directly accessed the *SMS for Life* website to view stock levels of AL and RDT in each facility within their district. The system highlighted stock-out alerts by depicting AL stock levels ≤30 and RDT ≤25 in red against the facility name (Figure 2B). Based on this information, DHMT members could apply corrective actions by redistributing stock from facilities with abundant stocks to those with stock below the minimum thresholds.

### Training of Participants

Training was staggered over three weeks from 22^nd^ August and 9^th^ September 2011 with health workers in Machakos district trained in the first week, Msambweni and Manga in the second and Ijara and Vihiga in the third week. One health worker from each health facility attended one day training during which their personal mobile phone numbers were registered into the *SMS for Life* system against their health facility names. Five different posters designed to mimic drug stores were used to train the health workers on how to count AL and RDT stocks. For each poster, the health workers received a test data request message and responded by counting the stock depicted; composing an SMS text message reply using system designed letter codes and sending the text to the designated short code number. Mistakes in counting and formatting were identified and corrected by health workers assisted by the trainers. A training manual and wall chart were given to each trained health worker to take to their health facility for reference. DHMT members were trained on how to access the *SMS for Life* system and view real-time stock data sent from health facilities in their district. Further coaching was provided on how to support health workers by identifying facilities with stock-out signals and those with stock that could be redistributed.

### Data Validation and Stock Movements

To assess accuracy of SMS reported data, health facility visits were conducted in the first and last month of the study. During each round of visits, a physical count of AL and RDT stock was made and compared with the SMS reported stock count of the same week adjusting for consumption from the day stock was reported to the day of visit (maximum 6 days). To assess response to stock-outs, facility based records were reviewed to extract data on dates and quantities of AL and RDTs issued to and received from other facilities during the intervention period. Similarly, KEMSA delivery notes retained at the health facilities were reviewed to extract data on dates and quantities of AL and RDT routinely delivered during the intervention period.

### Data Management and Analysis

Weekly stock data were extracted from the *SMS for Life* web database and analyzed in Excel and STATA, version 11 (Stata Corp, College Station, Texas). Data collected during the health facility visits were entered into ACCESS (Microsoft, USA) database and exported to STATA for analysis. Analysis was undertaken to evaluate response rates, SMS formatting error rates, accuracy of reported data, system use, stock-out trends and response to stock-out signals. Response rates were calculated as the mean number of correctly formatted text messages sent by health workers each week and over the 26 weeks of intervention. Similarly, error rates were calculated as the mean number of incorrectly formatted text messages sent each week and over the 26 weeks study period. Accuracy was assessed by calculating the percentage discrepancy between gold standard physical stock count made by the study team during the health facility visits and the health worker reported SMS data for the same week. A cut-off of +/−10% around the gold standard was applied when classifying health worker reported SMS as correct. This was done to account for minor discrepancies that may have occurred due to inaccurate recording of consumption of commodities between the time of SMS report and the facility visit. System use was measured as the absolute number of times the district managers accessed the system during the study, the average number of log-ins per week and the percentage of study weeks in which the system was accessed. Three stock-out indicators were measured: stock-out of all four AL packs, stock-out of one or more AL packs, and stock-out of each individual commodity (AL 6, AL 12, AL 18, AL 24 and RDT). Impact on stock-out was measured as the absolute percentage change of health facilities with stock-out of each parameter between the first (week 1) and last week (week 26) of the study. A descriptive account of observed stock-out trends was provided and compared to stock-out trends observed in a nationally representative sample of health facilities over the same duration as the study [14]. Finally, response to stock-outs was analyzed by counting the number of stock-out signals posted as alerts in the web-based database and relating them to data on inter-facility stock redistribution and KEMSA supplies collected during the health facility visits. The percentage of stock-out signals resolved through inter-facility redistribution of commodities undertaken by district managers and the proportion of stock-out signals resolved by regular KEMSA supplies were calculated.

### Ethical Approval

Ethical clearance for the study was obtained from the Kenya Medical Research Institute Ethical Review Committee; reference number SSC 2055. Written informed consent was obtained for all health workers who participated in the study. The ethical review board approved the consent procedure.

## Results

### Response and SMS Formatting Error Rates

**Figure 3:**
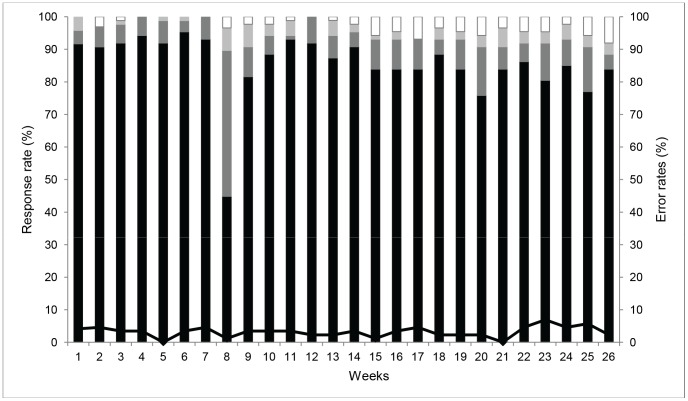
Weekly proportion of health facilities that responded to stock request messages and SMS formatting errors. Legend. Black bars show responses within 0–24 hrs; dark grey bars responses within 24–27 hrs (after reminder but within incentive period); light grey bars responses within 27 hrs-7 days (after the incentive period); white bars shows no responses, and black line shows SMS formatting errors.

Overall mean response rate during the 26 weeks study period was 97.1%. Most of the facilities (85.6%) responded within the reminder period (Thursday 2 pm to Friday 2 pm), 8.7% responded after the reminder message but still within the incentive period (2 pm–5 pm on Friday) and 2.8% sent the message after the incentive period (Friday 5 pm to Thursday 1 pm) (Figure 3). The high proportion of delayed responses observed in the week eight was due to network problems at the time of reporting. Mean response rates ranged from 93.4% in Machakos district to 100% in Vihiga district. Mean SMS formatting error rates across the five districts were 3.2%. Mean formatting error rates ranged from 2.4% in Msambweni to 5.6% in Manga district.

### System Usage

Over the 26 weeks study period, district managers accessed the *SMS for Life* system 1,037 times in 82% of the study weeks. Average number of log-ins was eight times per week. In Ijara and Manga districts, the managers accessed the system every week with a total of 362 (weekly mean 14) and 234 (weekly mean 9) log-ins respectively. Managers in Msambweni and Vihiga districts accessed the system in 69% of the study weeks with 219 (weekly mean 8) and 94 (weekly mean 4) log-ins respectively, while Machakos district had 128 log-ins (weekly mean 5) made in 73% of the study weeks. Finally, DOMC officers at the national level made 167 log-ins (weekly mean 6) accessing the system at least once in 22 of the 26 (85%) study weeks.

### Accuracy of Reported SMS Data

**Figure 4:**
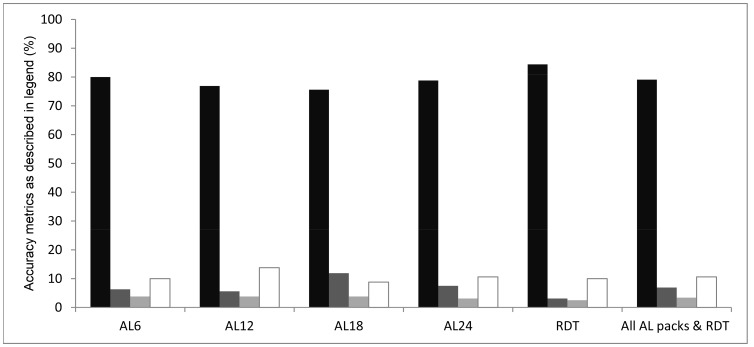
Accuracy of SMS reported stock parameters. Legend. Black bars show correctly reported stock counts (+/−0–10%); dark grey bars show counts with discrepancy +/−10–20%; light grey bars show counts with discrepancy +/−20–30%, and white bars show counts with >30% discrepancy.

Accuracy of SMS reported data was assessed at 86 and 74 facilities during the first and last month of the study respectively. Overall, 79.1% of all stock parameters were accurately reported with small variations between parameters (range 76% to 84%) (Figure 4). Accuracy of stock reports was similar at the beginning (81%) and at the end of the project (77%) as well as between districts, ranging from 78% in Msambweni and Manga to 85% in Ijara district. Notably, when commodity stock was zero and lower than 30 units, accuracy of reporting was higher with respectively 93% and 95% of stock counts correctly reported.

### AL Stock-out Trends and Responses to Stock-out Signals

**Figure 5:**
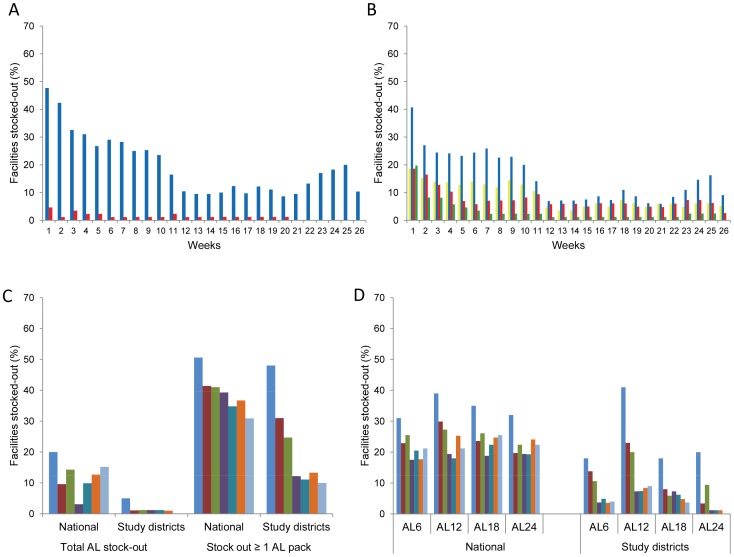
AL stock-out trends in study districts and stock-out comparisons with national trends. (A) Proportion of health facilities stocked out of all four AL packs and at least one AL pack over 26 weeks. Legend. Blue bars show stock-outs of at least one AL pack; red bars show stock-outs of all four AL packs. (B) Proportion of health facilities stocked out of AL 6, AL 12, AL 18 and AL 24 packs over 26 weeks. Legend. Yellow bars show stock-out of AL 6; blue bars stock-out of AL 12; red bars stock-out of AL 18 and green bars stock-out of AL 24 (C) Stock-out trends of all four AL packs and at least one AL pack in study districts compared to a nationally representative sample. Legend. The 7 consecutive bars show stock-outs in August, September, October, November, December, January and February. (D) Stock-out trends of AL 6, AL 12, AL 18 and AL 24 packs in study districts compared to a nationally representative sample. Legend. The 7 consecutive bars show stock-outs in August, September, October, November, December, January and February.

During the first week of the project, 47.7% of facilities were stocked out of one or more AL packs, most commonly AL 12 pack (41%) while stock‐outs of the other three packs were lower at 19‐20% (Figure 5 A–B). Five percent of facilities had total stock‐out of all four AL packs. Stock‐outs of individual AL packs declined gradually over time while total stock‐out of all four packs was eliminated by week 21 and sustained until the end of the 26 weeks monitoring period. Increase in stock-outs observed between week 21 and 25 was mainly due to increased stock-out of AL 12 pack reported in Msambweni district in that period. Comparing results between week 1 and 26, the percentage of facilities stocked‐out of one or more AL packs declined by 38%, total stock‐out of AL declined by 5%, while stock‐out declines for individual AL packs ranged from 14% for AL 6 to 32% for AL 12 pack. At the end of the monitoring period, respective stock-outs for AL 6, AL 12 and AL 18 packs were 5%, 9% and 3% while stock-out of AL 24 pack was entirely eliminated (Figure 5 A–B). Finally, in all five study districts, there was a decline in stock-outs of one or more AL packs ranging from 23% in Manga district to 75% in Vihiga district.

District managers responded to 44% of 176 stock-out alerts posted in the *SMS for Life* system by undertaking AL redistribution between facilities. A further 43% of AL stock-out signals were resolved by routine KEMSA distribution unrelated to the use of *SMS for Life* system while 13% of the alerts remained unresolved until the end of the monitoring period. Peripheral redistribution was the dominant response mechanism to stock-out signals in three districts (Ijara, Msambweni and Manga) (district range 60–77%). In the remaining two districts (Machakos and Vihiga), redistribution was rarely undertaken (8% and 7% respectively) and most signals were resolved by routine KEMSA supplies (58% and 93% respectively).

Stock-out declines of AL in the study areas were greater, sharper and more sustained than observed nationally in the same period (Figure 5C–D). Nationally, stock-out of one or more AL packs declined from 51% to 31% (20% decline) compared to a 38% decline in the study areas. Decline in total AL stock-out was similar between the national sample (20% to 15%) and study areas (5% to 0%). However, at the end of the monitoring period 15% of facilities in the national sample had total stock-out of AL as opposed to none in the study areas. Finally, declines in stock-outs of individual AL packs were lower in the national sample (9% to 18%) compared to declines in the study areas (14% to 32%) (Figure 5D). At the end of the monitoring period, national stock-outs for individual AL packs ranged from 21% for AL 6 and AL 12 pack to 26% for AL 18 pack (Figure 5D) while in the study districts, the stock-outs ranged from zero for AL 24 to 9% for AL 12 pack.

### RDT Stock-out Trends and Responses to Stock-out Signals

**Figure 6:**
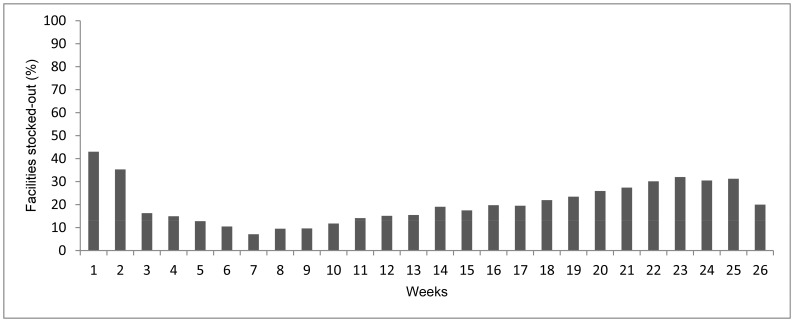
RDT stock-out trend over 26 weeks monitoring period.

Figure 6 presents RDT stock-out trends over the 26 weeks study period. Stock-outs of RDT declined from 43% in the first week to 20% in the last week of the project. Stock-outs declined rapidly after the start of the project reaching 7% in week seven. However, from week 11 stock-outs increased gradually reaching 32% in week 23 but dropped to 20% in the last week of the project. Of 103 RDT stock-out alerts signaled by the system, 73% were resolved by district managers through peripheral redistribution, 13% by KEMSA through the routine supply and 15% of signals remained unresolved. Peripheral redistribution was the dominant response action to RDT stock-out signals in all study districts (district range 56–100%). There was no resupply of RDTs in four of the five study districts, hence an increase in stock-outs was observed in the second part of the project.

## Discussion

The *SMS for Life* study in Kenya showed high weekly response rates (97%), low formatting error rates (3%) and reasonably high accuracy of reported stock count (79%) and stock-out data (93%). District managers frequently accessed the *SMS for Life* system and undertook corrective actions which contributed to elimination of total AL stock-out and substantial decline in stock-outs of individual AL packs (Figure 5A–B).

The high reporting and low formatting error rates observed in our study are consistent with 95% response rates and 7% error rates reported previously in Tanzania [15] thus confirming the simplicity of data reporting via text-messaging from the peripheral health facilities. We observed higher reporting rates than 85% found in a similar SMS study in Uganda [16]. However, unlike Kenyan and Tanzanian studies where health workers reporting SMS data received an incentive, the Ugandan results were obtained without the use of incentives. The Kenyan DOMC used incentives to optimize reliable measurement of facility stock-outs for which non-responses higher than 10% were deemed unsatisfactory and likely to introduce a substantial reporting bias. Given that impact on stock-outs has now been demonstrated, close monitoring of response rates without incentives coupled with corrective actions through routine district channels for underperforming facilities should be an integral component of potential scale up. Our study showed lower accuracy of reported data (79%) compared to 94% reported in Tanzania [15]. However, the Tanzanian health workers counted only unopened commodity boxes as opposed to counts of all individual AL packs in our study. We opted for counts of individual packs in order to align reporting procedures with routine logistic information systems in Kenya. As observed by study teams during the health facility visits, further improvements in the accuracy of reported data may be achieved by emphasizing during the training and supervisory visits that project trained health workers undertake actual physical stock count each time before sending the data.

We found that data on web-based *SMS for Life* platform were regularly accessed at the national level and by district managers. This shows that *SMS for Life* system is simple to use for district managers and highlights high visibility of real time data and its responsiveness potential. In Kenya, where only district managers can undertake immediate corrective action to mitigate stock-outs, high usage of the system is encouraging. The managers’ use of the system did not only encompass real-time viewing of the stock information but also acting upon detected stock-out alerts. District managers acted upon stock-out alerts by moving stock between facilities. The importance of these actions in study districts is even greater since this activity is not routinely done in Kenya and no additional resources or supportive supervision beyond the initial training was provided. Finally, while we have demonstrated district responses to simple stock-out signals defined as content of single commodity box, future work should explore whether more refined reallocation signals based on minimum and maximum stock levels can be reliably determined for individual facilities in Kenya.

RDT stock-out trends observed in this study brings an additional light to the use of *SMS for Life* system as a district tool to mitigate stock-outs. As opposed to AL routine distributions which are regular, although they often fail to supply the right drugs in the right quantities, RDT supplies had no resupply in four out of the five study districts. Therefore, despite a substantial and successful effort in reducing initial RDT stock-outs through peripheral redistribution, stocks in many areas were entirely depleted over time, thus precluding any corrective action by district managers. This inevitably resulted in a rebound of RDT stock-outs observed in the second part of the project. Lack of routine supply is probably the most detrimental factor affecting motivation of district managers to use the system and undertake corrective measures. We however believe that regular RDT distributions will be established over time in Kenya and that *SMS for Life* could be fully utilized to achieve its maximum impact.

A general limitation of SMS studies reporting trends and impact on commodity stocks is the absence of a randomized controlled design to account for temporal trends in non-intervention areas [15], [16]. To circumvent this limitation in our study, we compared results in the study districts with national AL stock-out trends in the same period. We indeed observed some declining trends nationally. However, national drug supply failed to eliminate the most detrimental total AL stock and declines in individual AL stock-out were substantially lower (Figure 5C–D) compared to the study districts. This finding, together with data on corrective measures undertaken by district managers to address stock-outs in response to the use of the web-based system, gives us the confidence that *SMS for Life* substantially contributed to observed AL stock-out reductions in the study districts.

Finally, our study contributes to a limited number of peer-reviewed reports on the use of SMS technology for data reporting within malaria control in Africa. Disease surveillance has been the topic of reporting in Zambia [17], [18], Madagascar [19], [20], Uganda [16] while only two studies in Tanzania [15] and Uganda [16] have reported on use of SMS for commodity monitoring. All the studies reported were pilot projects or national sentinel sites which have demonstrated the feasibility of real-time data reporting using basic, personal mobile phones. The studies have also achieved high reporting rates highlighting the potential utility of SMS reporting for malaria control managers. Our study, as well as the study in Tanzania [15], went a step further to show that SMS reporting under routine conditions is able not only to ensure visibility of data for potential action but indeed triggered corrective responses at district level. These corrective actions resulted in direct benefits for the reporting health workers and the patients they serve.

### Conclusions

*SMS for Life* study in Kenya has demonstrated that use of simple SMS technology ensured high reporting rates of reasonably accurate, real-time facility stock data that were used by district managers to undertake corrective actions and reduce stock-outs of antimalarial commodities. Future work on stock monitoring via SMS should focus on demonstrating the effectiveness of SMS interventions on a larger scale and in the absence of health worker incentives. Inclusion of other life saving therapies within SMS reports should also be explored.
